# Crystal structure of bis­{μ-2-meth­oxy-6-[(methyl­imino)­meth­yl]phenolato}bis­({2-meth­oxy-6-[(methyl­imino)­meth­yl]phenolato}nickel(II)) involving different coordination modes of the same Schiff base ligand

**DOI:** 10.1107/S2056989019004766

**Published:** 2019-04-12

**Authors:** Olga Yu. Vassilyeva, Vladimir N. Kokozay, Brian W. Skelton

**Affiliations:** a Department of Chemistry, Taras Shevchenko National University of Kyiv, 64/13 Volodymyrska Street, Kyiv 01601, Ukraine; b School of Molecular Sciences, M310, University of Western Australia, Perth, WA 6009, Australia

**Keywords:** crystal structure, Ni^II^ dimer, Schiff base ligand, *o*-vanillin, methyl­amine

## Abstract

The structure of the title compound, [Ni_2_(C_9_H_10_NO_2_)_4_], is built up by discrete centrosymmetric dimers. Two nitro­gen and three oxygen atoms of two Schiff base ligands singly deprotonated at the phenolate site form a square-pyramidal environment for each metal atom. The ligands are bonded differently to the metal centre: one of the phenolic O atoms is bound to one nickel atom, whereas another bridges the two metal atoms to form the dimer. The Ni—N/O distances fall in the range 1.8965 (13)–1.9926 (15) Å, with the Ni—N bonds being slightly longer; the fifth contact of the metal to the bridging phenolate oxygen atom is substanti­ally elongated [2.533 (1) Å]. A similar coordination geometry was observed in the isomorphous Cu analogue previously reported by us [Sydoruk *et al.* (2013 ▸). *Acta Cryst*. E**69**, m551–m552]. In the crystal, the [Ni_2_ *L*_4_] mol­ecules form sheets parallel to the *ab* plane with the polar meth­oxy groups protruding into the inter­sheet space and keeping the sheets apart. Within a sheet, the mol­ecules are stacked relative to each other in such a way that the Ni_2_O_2_ planes of neighbouring mol­ecules are orthogonal.

## Abstract 2

Different coordination modes of the same Schiff base ligand enable formation of the dimeric Ni^II^ complex. The phenolato-bridged metal centres are further apart in the Ni dimer compared to the isomorphous Cu compound.

## Chemical context   

The title compound, [Ni_2_(C_9_H_10_NO_2_)_4_], **1**, has been synthesized as part of our long-term research on Schiff base metal complexes aimed at the preparation of mono- and heterometallic compounds of various compositions and structures, and the investigation of their potential applications. In these studies, we use direct synthesis of coordination compounds based on a spontaneous self-assembly in solution, in which the metal (or one of the metals in the case of heterometallic complexes) is introduced as a fine powder (zerovalent state) and oxidized by aerial di­oxy­gen during the synthesis (Buvaylo *et al.*, 2005 ▸, 2012 ▸; Kokozay *et al.*, 2018 ▸).

The multidentate ligand 2-meth­oxy-6-[(methyl­imino)­meth­yl]phenol, H*L*, derived from 2-hy­droxy-3-meth­oxy-benzaldehyde (*o*-vanillin) and methyl­amine shows various connectivity fashions and can generate mono- and polymetallic complexes. The meth­oxy group plays an essential role in the coordination abilities of the Schiff base (Andruh, 2015 ▸). The singly deprotonated H*L* ligand has been shown to act as a multidentate linker between seven metal centres affording [*M*_7_] assemblies, where *M* is a divalent Ni, Zn, Co or Mn ion (Meally *et al.*, 2010 ▸, 2012 ▸; Zhang *et al.*, 2010 ▸). The octa­hedral metal atoms in the hepta­nuclear cores are additionally supported by μ_3_-bridging OH^−^ or MeO^−^ groups that link the central metal atom to the six peripheral ones. Of heterometallic examples with H*L*, only four 1*s*–3*d* structures of Na/*M* (*M* = Fe, Ni) complexes have been reported (Meally *et al.*, 2013 ▸).
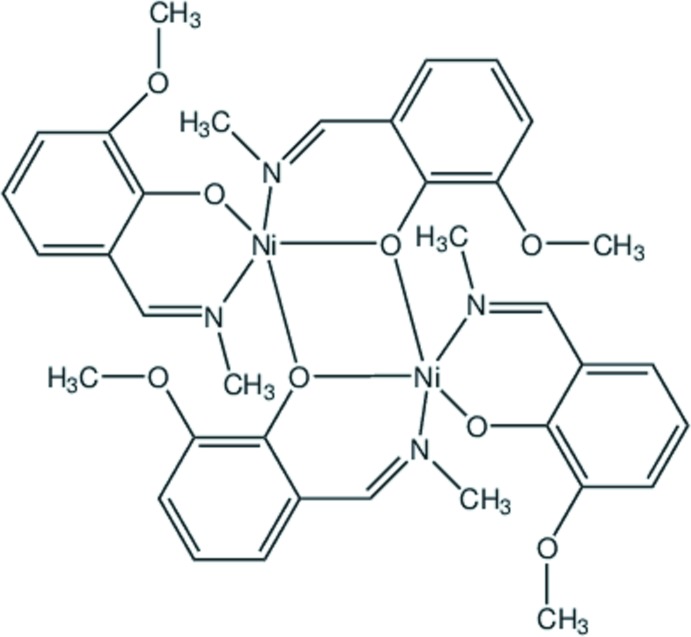


Our research efforts in the field have yielded novel heterometallic dinuclear Co^III^/Cd and Co^III^/Zn complexes bearing H*L* along with the ‘parent’ mononuclear complex Co*L*_3_·DMF (DMF = *N*,*N*-di­methyl­formamide; Nesterova *et al.*, 2018 ▸, 2019 ▸; Vassilyeva *et al.*, 2018 ▸). Their catalytic activity in stereospecific alkanes oxidation with *m*-chloro­perbenzoic acid as an oxidant has been studied in detail. A comparison of the catalytic behaviours of the hetero- and monometallic analogues provided further insight into the origin of stereoselectivity of the oxidation of C—H bonds. In the syntheses, the condensation reaction between *o*-vanillin and CH_3_NH_2_·HCl was utilized without isolation of the resulting Schiff base. In the present work, the title compound was isolated in an attempt to prepare a heterometallic Ni/Sn complex with H*L* in the reaction of nickel powder and SnCl_2_·2H_2_O, with the Schiff base formed *in situ* in a methanol/DMF mixture in a 1:1:2 molar ratio. Similarly to the synthesis of Co*L*_3_·DMF (Nesterova *et al.*, 2018 ▸), H*L* does not enable the formation of a heterometallic Sn-containing species, in contrast to its compartmental analogues 3-*R*-salicyl­aldehyde-ethyl­enedi­amine (*R* = meth­oxy-, eth­oxy-), H*L*′, that afford heterometallic, diphenoxido-bridged, dinuclear Cu^II^Sn^II^ cations [Cu*L*′SnCl]^+^ (Hazra *et al.*, 2016 ▸).

## Structural commentary   

**Figure 1:**
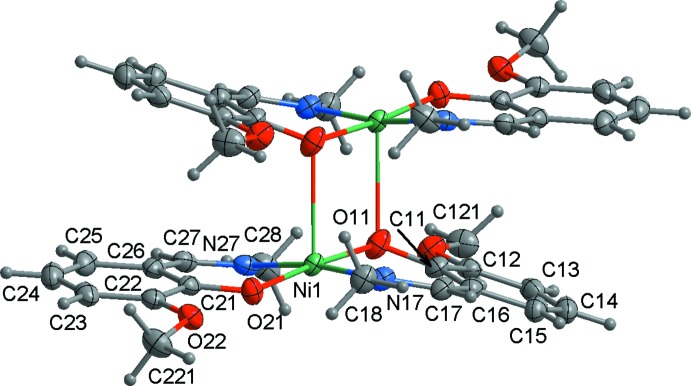
The mol­ecular structure of the title compound, showing the atom-numbering scheme for the asymmetric unit. Non-H atoms are shown with displacement ellipsoids drawn at the 50% probability level.

The mol­ecular structure of **1** exists as a centrosymmetric dimer [Ni_2_ *L*_4_] (Fig. 1 ▸). The nickel atom is five-coordinate with two nitro­gen and three oxygen atoms of two, singly deprotonated at the phenolate site Schiff base ligands. The ligands are bonded differently to the metal atoms: the phenolic oxygen atom O21 is bound to one nickel atom, whereas O11 bridges the two metal centres and forms the dimer.

The Ni—N bonds are somewhat longer than the shortest Ni—O distances (Table 1 ▸) while the fifth contact of the metal to the bridging oxygen atom is substanti­ally elongated. The *cis* angles at the nickel atom are in the range 87.57 (6)–91.09 (6)°, with the two *trans* angles being 170.92 (6) and 175.66 (6)° (Table 1 ▸). The angular structural index parameter, τ = (β – α)/60, evaluated from the two largest angles (α < β) in the five-coordinate geometry is 0.08 compared with ideal values of 1 for an equilateral bipyramid and 0 for a square pyramid. Hence, the nickel coordination polyhedron in **1** is a square pyramid with minimal distortion. The apical position of the coordination sphere is occupied by the bridging phenolate oxygen O11(1 − *x*, 1 − *y*, 1 − *z*) with a bridging angle of 101.44 (2)°.

We reported a similar coordination geometry for the isomorphous Cu analogue [Cu_2_ *L*_4_; Sydoruk *et al.*, 2013 ▸]. The main difference between the two structures is the proximity of the metal centres in the dimers, which are further apart in the Ni complex compared to the Cu compound. The Ni⋯Ni distance is 3.4638 (4) compared to the Cu⋯Cu separation of 3.3737 (2) Å. In addition, the Cu—O11(1 − *x*, 1 − *y*, 1 − *z*) contact in [Cu_2_ *L*_4_] is shorter [2.4329 (7) Å].

## Supra­molecular features   

**Figure 2:**
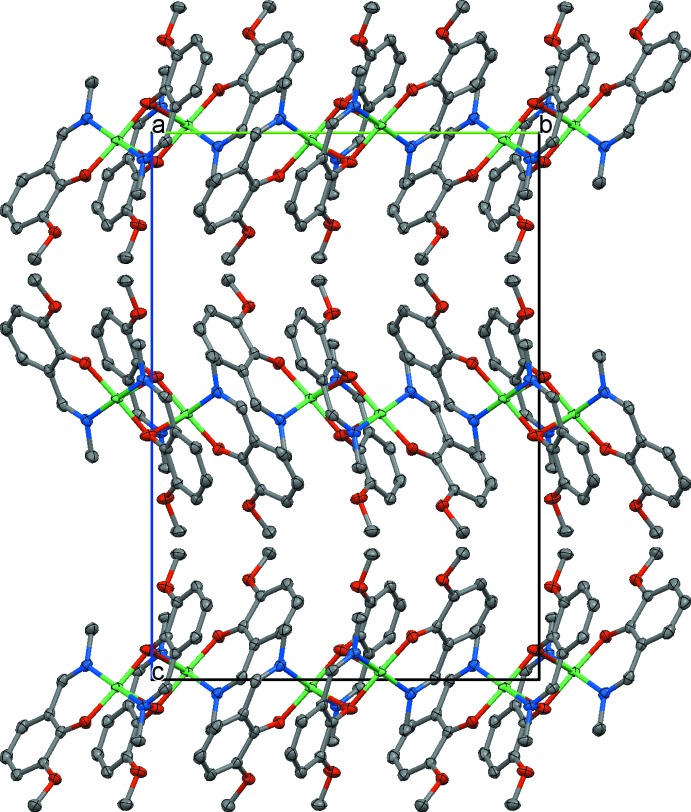
Crystal packing of **1** showing sheets of [Ni_2_ *L*_4_] mol­ecules parallel to the *ab* plane. H atoms are not shown.

**Figure 3:**
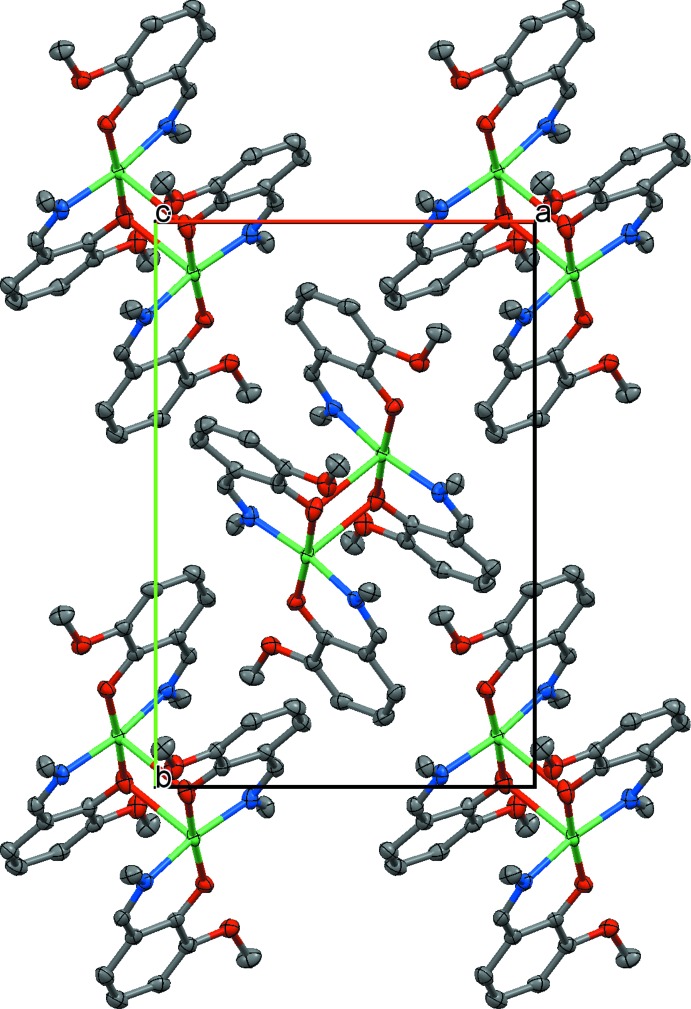
Fragment of the sheet of [Ni_2_ *L*_4_] mol­ecules viewed down the *c* axis showing the orthogonal packing of neighboring dimers. H atoms are not shown.

There are no significant inter­molecular inter­actions between the dimers in the crystal lattice. Classical hydrogen-bonding inter­actions are absent in **1**. The mol­ecules form sheets parallel to the *ab* plane with the non-coordinating polar meth­oxy groups protruding into the inter­sheet space and keeping the sheets apart (Fig. 2 ▸). Within a sheet, the mol­ecules pack relative to each other in such a way that neighbouring Ni_2_O_2_ planes are orthogonal (Fig. 3 ▸). The minimum Ni⋯Ni separations inside a sheet and between adjacent sheets are about 7.099 and 11.374 Å, respectively. The C—H⋯O inter­action between C28—H28*A* and O22(*x* + 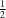, −*y* + 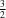, −*z* + 1) [C28—H28*A* = 0.98 Å, H28*A*⋯O22 = 2.57 Å, C28⋯O22 = 3.449 (2) Å and C28—H28*A*⋯O22 = 150°] is very weak.

## Database survey   

A search in the Cambridge Structural Database (CSD; Groom *et al.*, 2016 ▸) for H*L* and its complexes *via* the WebCSD inter­face in March 2019 reveals that 39 original crystal structures, including the structure of the ligand itself, have been reported. Polynuclear complexes constitute the majority of the structures with 17 examples of [*M* ^II^_7_] (*M* = Mn, Co, Ni, Zn) assemblies featuring planar hexa­gonal disc-like cores and three examples of dimeric (Cu_2_) and tetra­meric complexes with the cubane- (Mn_4_) or open-cubane type cores (Co_4_). The singly deprotonated H*L* ligand evidently encourages the formation of polynuclear metal complexes only with assistance from other bridging ligands. The integrity of the hepta- [*M* ^II^_7_ *L*_6_] and tetra­nuclear [Mn_4_ *L*_3_], [Co_4_ *L*_2_] polymetallics is secured by μ_3_-bridging OH^−^/MeO^−^ groups and other ligands, respectively. A higher metal-to-ligand ratio (1:2 and 1:3) in the absence of bridging ligands stimulates the formation of mononuclear complexes, as evidenced by the 10 structures with mol­ecular (Mn, Co and Pt) or polymeric (Mn) arrangements in the crystal lattice. The four heterometallic examples with H*L* published by others are limited to Na/*M* (*M* = Fe, Ni) complexes whose formation was induced by the use of sodium salts and/or NaOH in the synthesis. The 3*d*–3*d*/4*d* heterometallics recently reported by our group are based on the neutral Co^III^ *L*_3_ species with the metal centre in a *mer* configuration that acts as a metalloligand to Zn^2+^/Cd^2+^ ions, generating [Co*ML*_3_Cl_2_]·Solv (Solv = H_2_O, CH_3_OH) complexes.

## Synthesis and crystallization   

*o*-Vanillin (0.3 g, 2.0 mmol) in 10 mL of methanol was stirred with CH_3_NH_2_·HCl (0.14 g, 2.0 mmol) in the presence of di­methyl­amino­ethanol (0.1 mL) in a 50 mL conical flask at 333 K for half an hour. SnCl_2_·2H_2_O (0.23 g, 1.0 mmol) dissolved in 10 mL of DMF and Ni powder (0.06 g, 1.0 mmol) were added to the resulting yellow solution of the preformed Schiff base. The mixture gradually turned brown while it was magnetically stirred at 333 K to achieve dissolution of the nickel (2 h; adhesion of a small fraction of the metal particles to the stirring bar precluded complete dissolution of the metal powder). The resultant brown solution was filtered and left to stand at room temperature. Dark-brown, almost black, prisms of **1** formed in two weeks. They were filtered off, washed with dry Pr^i^OH and dried in air. Yield (based on Ni): 31%. Analysis calculated for C_36_H_40_N_4_Ni_2_O_8_ (774.14): C 55.86, H 5.21, N 7.24%. Found: C 55.62, H 5.33, N 7.11%.

**Figure 4:**
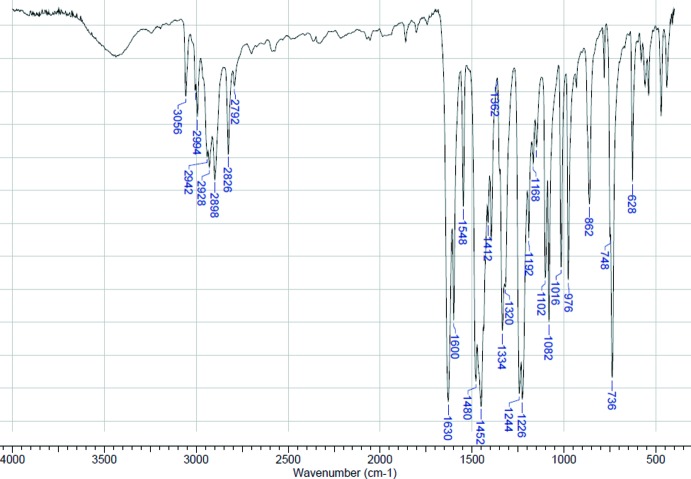
IR spectrum of **1** in a KBr pellet.

A broad band centered at about 3440 cm^−1^ in the IR spectrum of **1** may be due to adsorbed water mol­ecules (Fig. 4 ▸). Several bands arising above and below 3000 cm^−1^ are assigned to aromatic =CH and alkyl –CH stretching, respectively. The characteristic ν(C=N) absorption of the Schiff base which appears at 1634 cm^−1^ as a strong intense band in the IR spectrum of H*L* (Nesterova *et al.*, 2018 ▸) is detected at 1630 cm^−1^ in the spectrum of **1**. A number of sharp and intense bands are observed in the aromatic ring stretching (1600–1400 cm^−1^) and C—H out-of-plane bending regions (800–700 cm^−1^).

## Refinement   

Crystal data, data collection and structure refinement details are summarized in Table 2 ▸. Hydrogen atoms were placed at idealized positions and refined using a riding model: C—H = 0.95 Å with *U*_iso_(H) = 1.2*U*_eq_(C) for CH, 0.98 Å and 1.5*U*_eq_(C) for CH_3_.

## Supplementary Material

Crystal structure: contains datablock(s) I, global. DOI: 10.1107/S2056989019004766/lh5898sup1.cif

Structure factors: contains datablock(s) I. DOI: 10.1107/S2056989019004766/lh5898Isup2.hkl

CCDC reference: 1908788

Additional supporting information:  crystallographic information; 3D view; checkCIF report

## Figures and Tables

**Table 1:** Selected geometric parameters (Å, °)

Ni1—O21	1.8965 (13)	Ni1—N17	1.9926 (15)
Ni1—O11	1.9135 (14)	Ni1—O11^i^	2.5326 (14)
Ni1—N27	1.9783 (15)		
			
O21—Ni1—O11	175.66 (6)	O11—Ni1—N17	90.70 (6)
O21—Ni1—N27	91.09 (6)	N27—Ni1—N17	170.92 (6)
O11—Ni1—N27	90.00 (6)	Ni1—O11—Ni1^i^	101.44 (2)
O21—Ni1—N17	87.57 (6)		

Symmetry code: (i) 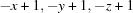.

**Table 2:** Experimental details

Crystal data	
Chemical formula	[Ni_2_(C_9_H_10_NO_2_)_4_]
*M*_r_	774.14
Crystal system, space group	Orthorhombic, *P* *b* *c* *a*
Temperature (K)	100
*a*, *b*, *c* (Å)	10.2301 (2), 15.2456 (3), 21.5426 (5)
*V* (Å^3^)	3359.87 (12)
*Z*	4
Radiation type	Mo *K*α
μ (mm^−1^)	1.18
Crystal size (mm)	0.37 × 0.27 × 0.23

Data collection	
Diffractometer	Oxford Diffraction Xcalibur
Absorption correction	Analytical (*CrysAlis PRO*; Rigaku OD, 2015 ▸)
*T*_min_, *T*_max_	0.816, 0.87
No. of measured, independent and observed [*I* > 2σ(*I*)] reflections	20556, 5548, 4332
*R*_int_	0.041
(sin θ/λ)_max_ (Å^−1^)	0.747

Refinement	
*R*[*F* ^2^ > 2σ(*F* ^2^)], *wR*(*F* ^2^), *S*	0.041, 0.088, 1.03
No. of reflections	5548
No. of parameters	230
H-atom treatment	H-atom parameters constrained
Δρ_max_, Δρ_min_ (e Å^−3^)	0.89, −0.61

Computer programs: *CrysAlis PRO* (Rigaku OD, 2015 ▸), *SHELXT* (Sheldrick, 2015 ▸a), *SHELXL2014* (Sheldrick, 2015 ▸b), *DIAMOND* (Brandenburg, 1999 ▸) and *Mercury* (Macrae *et al.*, 2006 ▸) and *WinGX* (Farrugia, 2012 ▸).

## supplementary crystallographic information

### Crystal data

**Table:**

[Ni_2_(C_9_H_10_NO_2_)_4_]	*F*(000) = 1616
*M**_r_* = 774.14	*D*_x_ = 1.53 Mg m^−^^3^
Orthorhombic, *P**b**c**a*	Mo *K*α radiation, λ = 0.71073 Å
Hall symbol: -P 2ac 2ab	Cell parameters from 6602 reflections
*a* = 10.2301 (2) Å	θ = 2.6–31.7°
*b* = 15.2456 (3) Å	µ = 1.18 mm^−^^1^
*c* = 21.5426 (5) Å	*T* = 100 K
*V* = 3359.87 (12) Å^3^	Prism, black
*Z* = 4	0.37 × 0.27 × 0.23 mm

### Data collection

**Table:**

Oxford Diffraction Xcalibur diffractometer	5548 independent reflections
Radiation source: fine-focus sealed X-ray tube, Enhance (Mo) X-ray Source	4332 reflections with *I* > 2σ(*I*)
Graphite monochromator	*R*_int_ = 0.041
Detector resolution: 16.0009 pixels mm^-1^	θ_max_ = 32.1°, θ_min_ = 2.6°
ω scans	*h* = −15→12
Absorption correction: analytical (CrysAlis PRO; Rigaku OD, 2015)	*k* = −22→22
*T*_min_ = 0.816, *T*_max_ = 0.87	*l* = −29→32
20556 measured reflections	

### Refinement

**Table:**

Refinement on *F*^2^	0 restraints
Least-squares matrix: full	Hydrogen site location: inferred from neighbouring sites
*R*[*F*^2^ > 2σ(*F*^2^)] = 0.041	H-atom parameters constrained
*wR*(*F*^2^) = 0.088	*w* = 1/[σ^2^(*F*_o_^2^) + (0.0253*P*)^2^ + 2.4148*P*] where *P* = (*F*_o_^2^ + 2*F*_c_^2^)/3
*S* = 1.03	(Δ/σ)_max_ = 0.002
5548 reflections	Δρ_max_ = 0.89 e Å^−^^3^
230 parameters	Δρ_min_ = −0.61 e Å^−^^3^

### Special details

**Table:**

Geometry. All esds (except the esd in the dihedral angle between two l.s. planes) are estimated using the full covariance matrix. The cell esds are taken into account individually in the estimation of esds in distances, angles and torsion angles; correlations between esds in cell parameters are only used when they are defined by crystal symmetry. An approximate (isotropic) treatment of cell esds is used for estimating esds involving l.s. planes.
Refinement. Three low theta reflections, considered to be partly hidden by the beam stop were omitted from the refnement.

### Fractional atomic coordinates and isotropic or equivalent isotropic displacement parameters (Å^2^)

**Table:**

	*x*	*y*	*z*	*U*_iso_*/*U*_eq_	
Ni1	0.40070 (2)	0.58979 (2)	0.51420 (2)	0.01932 (7)	
C11	0.32402 (17)	0.46376 (12)	0.42138 (9)	0.0242 (4)	
O11	0.41820 (12)	0.50907 (10)	0.44669 (6)	0.0315 (3)	
C12	0.33944 (18)	0.43535 (12)	0.35897 (9)	0.0250 (4)	
O12	0.45143 (13)	0.46517 (10)	0.33106 (6)	0.0330 (3)	
C121	0.4805 (2)	0.43159 (15)	0.27123 (9)	0.0363 (5)	
H12A	0.4893	0.3677	0.2735	0.054*	
H12B	0.5626	0.4572	0.2563	0.054*	
H12C	0.4097	0.4467	0.2425	0.054*	
C13	0.2460 (2)	0.38443 (12)	0.33055 (9)	0.0292 (4)	
H13	0.2578	0.3661	0.2888	0.035*	
C14	0.1331 (2)	0.35947 (13)	0.36323 (10)	0.0331 (4)	
H14	0.0691	0.3238	0.3436	0.04*	
C15	0.11502 (19)	0.38630 (13)	0.42312 (10)	0.0295 (4)	
H15	0.0384	0.3692	0.4449	0.035*	
C16	0.20898 (17)	0.43913 (12)	0.45289 (9)	0.0243 (4)	
C17	0.18436 (17)	0.46528 (12)	0.51630 (9)	0.0253 (4)	
H17	0.1124	0.4384	0.5366	0.03*	
N17	0.25052 (14)	0.52145 (10)	0.54782 (7)	0.0252 (3)	
C18	0.21030 (18)	0.53585 (14)	0.61258 (9)	0.0292 (4)	
H18A	0.1355	0.4981	0.6223	0.044*	
H18B	0.1854	0.5974	0.6182	0.044*	
H18C	0.2832	0.5216	0.6404	0.044*	
C21	0.44702 (17)	0.73584 (11)	0.59655 (9)	0.0231 (3)	
O21	0.37097 (12)	0.67232 (8)	0.57853 (6)	0.0262 (3)	
C22	0.41501 (17)	0.78091 (12)	0.65278 (9)	0.0247 (4)	
O22	0.30458 (13)	0.75045 (9)	0.68192 (6)	0.0277 (3)	
C221	0.2536 (2)	0.80316 (14)	0.73071 (10)	0.0360 (5)	
H22A	0.3158	0.8041	0.7653	0.054*	
H22B	0.1703	0.7786	0.7449	0.054*	
H22C	0.2398	0.8631	0.7156	0.054*	
C23	0.49076 (19)	0.84910 (12)	0.67430 (9)	0.0296 (4)	
H23	0.4677	0.878	0.7118	0.036*	
C24	0.6015 (2)	0.87618 (14)	0.64127 (10)	0.0337 (4)	
H24	0.6537	0.9231	0.6564	0.04*	
C25	0.63413 (19)	0.83488 (13)	0.58706 (10)	0.0311 (4)	
H25	0.7094	0.8534	0.5648	0.037*	
C26	0.55760 (17)	0.76490 (12)	0.56357 (9)	0.0246 (4)	
C27	0.58830 (17)	0.73171 (12)	0.50282 (9)	0.0257 (4)	
H27	0.6594	0.7585	0.4817	0.031*	
N27	0.52852 (14)	0.66906 (10)	0.47419 (7)	0.0249 (3)	
C28	0.5641 (2)	0.65577 (14)	0.40857 (9)	0.0334 (4)	
H28A	0.6369	0.6946	0.3977	0.05*	
H28B	0.4887	0.6692	0.3822	0.05*	
H28C	0.5903	0.5946	0.4022	0.05*	

### Atomic displacement parameters (Å^2^)

**Table:**

	*U*^11^	*U*^22^	*U*^33^	*U*^12^	*U*^13^	*U*^23^
Ni1	0.01748 (10)	0.02415 (12)	0.01631 (11)	−0.00316 (8)	0.00124 (8)	0.00016 (9)
C11	0.0218 (8)	0.0283 (9)	0.0225 (9)	0.0008 (7)	−0.0043 (7)	0.0013 (7)
O11	0.0250 (6)	0.0462 (8)	0.0233 (7)	−0.0070 (6)	0.0005 (5)	−0.0058 (6)
C12	0.0260 (8)	0.0253 (8)	0.0235 (9)	0.0055 (7)	−0.0028 (7)	0.0005 (7)
O12	0.0292 (7)	0.0466 (9)	0.0232 (7)	0.0010 (6)	0.0023 (6)	−0.0065 (6)
C121	0.0437 (12)	0.0427 (12)	0.0225 (9)	0.0090 (9)	0.0033 (9)	−0.0015 (9)
C13	0.0371 (10)	0.0243 (9)	0.0263 (10)	0.0040 (7)	−0.0050 (8)	−0.0022 (8)
C14	0.0391 (11)	0.0263 (9)	0.0340 (11)	−0.0055 (8)	−0.0094 (9)	−0.0005 (8)
C15	0.0298 (9)	0.0275 (9)	0.0311 (10)	−0.0062 (7)	−0.0043 (8)	0.0032 (8)
C16	0.0254 (8)	0.0239 (8)	0.0235 (9)	−0.0002 (7)	−0.0036 (7)	0.0021 (7)
C17	0.0222 (8)	0.0292 (9)	0.0246 (9)	−0.0029 (7)	−0.0016 (7)	0.0057 (8)
N17	0.0221 (7)	0.0320 (8)	0.0215 (7)	−0.0002 (6)	0.0008 (6)	0.0027 (7)
C18	0.0282 (9)	0.0369 (10)	0.0226 (9)	−0.0044 (8)	0.0052 (8)	0.0008 (8)
C21	0.0227 (8)	0.0196 (8)	0.0270 (9)	0.0020 (6)	−0.0019 (7)	0.0053 (7)
O21	0.0263 (6)	0.0241 (6)	0.0283 (7)	−0.0038 (5)	0.0065 (5)	−0.0033 (6)
C22	0.0272 (9)	0.0218 (8)	0.0252 (9)	0.0022 (7)	−0.0024 (7)	0.0041 (7)
O22	0.0313 (7)	0.0264 (6)	0.0254 (7)	0.0015 (5)	0.0054 (6)	−0.0020 (6)
C221	0.0481 (12)	0.0317 (10)	0.0282 (10)	0.0063 (9)	0.0075 (10)	−0.0017 (9)
C23	0.0392 (10)	0.0225 (9)	0.0272 (10)	0.0011 (8)	−0.0074 (8)	0.0022 (8)
C24	0.0386 (11)	0.0272 (9)	0.0353 (11)	−0.0077 (8)	−0.0101 (9)	0.0045 (9)
C25	0.0279 (9)	0.0288 (9)	0.0367 (11)	−0.0065 (7)	−0.0041 (8)	0.0099 (9)
C26	0.0236 (8)	0.0211 (8)	0.0293 (9)	0.0004 (6)	−0.0025 (7)	0.0065 (7)
C27	0.0209 (8)	0.0231 (8)	0.0331 (10)	0.0026 (6)	0.0033 (7)	0.0060 (8)
N27	0.0245 (7)	0.0233 (7)	0.0270 (8)	0.0044 (6)	0.0039 (6)	0.0055 (6)
C28	0.0402 (11)	0.0292 (10)	0.0307 (10)	0.0035 (8)	0.0129 (9)	0.0047 (8)

### Geometric parameters (Å, º)

**Table:**

Ni1—O21	1.8965 (13)	C18—H18B	0.98
Ni1—O11	1.9135 (14)	C18—H18C	0.98
Ni1—N27	1.9783 (15)	C21—O21	1.302 (2)
Ni1—N17	1.9926 (15)	C21—C26	1.407 (2)
Ni1—O11^i^	2.5326 (14)	C21—C22	1.431 (3)
C11—O11	1.305 (2)	C22—O22	1.373 (2)
C11—C16	1.410 (3)	C22—C23	1.377 (3)
C11—C12	1.421 (3)	O22—C221	1.422 (2)
C12—O12	1.371 (2)	C221—H22A	0.98
C12—C13	1.375 (3)	C221—H22B	0.98
O12—C121	1.418 (2)	C221—H22C	0.98
C121—H12A	0.98	C23—C24	1.400 (3)
C121—H12B	0.98	C23—H23	0.95
C121—H12C	0.98	C24—C25	1.368 (3)
C13—C14	1.405 (3)	C24—H24	0.95
C13—H13	0.95	C25—C26	1.417 (3)
C14—C15	1.366 (3)	C25—H25	0.95
C14—H14	0.95	C26—C27	1.438 (3)
C15—C16	1.409 (3)	C27—N27	1.291 (2)
C15—H15	0.95	C27—H27	0.95
C16—C17	1.445 (3)	N27—C28	1.474 (2)
C17—N17	1.286 (2)	C28—H28A	0.98
C17—H17	0.95	C28—H28B	0.98
N17—C18	1.471 (2)	C28—H28C	0.98
C18—H18A	0.98		
			
O21—Ni1—O11	175.66 (6)	N17—C18—H18C	109.5
O21—Ni1—N27	91.09 (6)	H18A—C18—H18C	109.5
O11—Ni1—N27	90.00 (6)	H18B—C18—H18C	109.5
O21—Ni1—N17	87.57 (6)	O21—C21—C26	124.35 (17)
O11—Ni1—N17	90.70 (6)	O21—C21—C22	118.23 (16)
N27—Ni1—N17	170.92 (6)	C26—C21—C22	117.40 (17)
Ni1—O11—Ni1^i^	101.44 (2)	C21—O21—Ni1	128.01 (12)
O11—C11—C16	123.79 (17)	O22—C22—C23	124.36 (18)
O11—C11—C12	118.34 (16)	O22—C22—C21	114.39 (16)
C16—C11—C12	117.84 (17)	C23—C22—C21	121.25 (17)
C11—O11—Ni1	126.08 (12)	C22—O22—C221	116.62 (15)
O12—C12—C13	124.95 (18)	O22—C221—H22A	109.5
O12—C12—C11	113.97 (16)	O22—C221—H22B	109.5
C13—C12—C11	121.06 (18)	H22A—C221—H22B	109.5
C12—O12—C121	116.98 (16)	O22—C221—H22C	109.5
O12—C121—H12A	109.5	H22A—C221—H22C	109.5
O12—C121—H12B	109.5	H22B—C221—H22C	109.5
H12A—C121—H12B	109.5	C22—C23—C24	120.45 (19)
O12—C121—H12C	109.5	C22—C23—H23	119.8
H12A—C121—H12C	109.5	C24—C23—H23	119.8
H12B—C121—H12C	109.5	C25—C24—C23	119.73 (19)
C12—C13—C14	120.08 (18)	C25—C24—H24	120.1
C12—C13—H13	120	C23—C24—H24	120.1
C14—C13—H13	120	C24—C25—C26	121.09 (19)
C15—C14—C13	120.23 (18)	C24—C25—H25	119.5
C15—C14—H14	119.9	C26—C25—H25	119.5
C13—C14—H14	119.9	C21—C26—C25	120.06 (18)
C14—C15—C16	120.59 (19)	C21—C26—C27	121.62 (17)
C14—C15—H15	119.7	C25—C26—C27	118.00 (17)
C16—C15—H15	119.7	N27—C27—C26	126.29 (17)
C15—C16—C11	120.18 (18)	N27—C27—H27	116.9
C15—C16—C17	117.99 (17)	C26—C27—H27	116.9
C11—C16—C17	121.82 (16)	C27—N27—C28	116.31 (16)
N17—C17—C16	126.25 (17)	C27—N27—Ni1	123.84 (13)
N17—C17—H17	116.9	C28—N27—Ni1	119.81 (13)
C16—C17—H17	116.9	N27—C28—H28A	109.5
C17—N17—C18	116.94 (16)	N27—C28—H28B	109.5
C17—N17—Ni1	124.21 (13)	H28A—C28—H28B	109.5
C18—N17—Ni1	118.85 (12)	N27—C28—H28C	109.5
N17—C18—H18A	109.5	H28A—C28—H28C	109.5
N17—C18—H18B	109.5	H28B—C28—H28C	109.5
H18A—C18—H18B	109.5		

Symmetry code: (i) −*x*+1, −*y*+1, −*z*+1.

### Hydrogen-bond geometry (Å, º)

**Table:**

*D*—H···*A*	*D*—H	H···*A*	*D*···*A*	*D*—H···*A*
C28—H28*A*···O22^ii^	0.98	2.57	3.449 (2)	150
C28—H28*A*···O22^ii^	0.98	2.57	3.449 (2)	150
C28—H28*C*···N17^i^	0.98	2.63	3.432 (3)	139

Symmetry codes: (i) −*x*+1, −*y*+1, −*z*+1; (ii) *x*+1/2, −*y*+3/2, −*z*+1.
